# Supplementary figures and images for: High crossreactivity of human T cell responses between Lassa virus lineages
Source: PLoS Pathog. 2020 Mar 6;16(3):e1008352. doi: 10.1371/journal.ppat.1008352 (PMC7080273; doi:10.1371/journal.ppat.1008352)

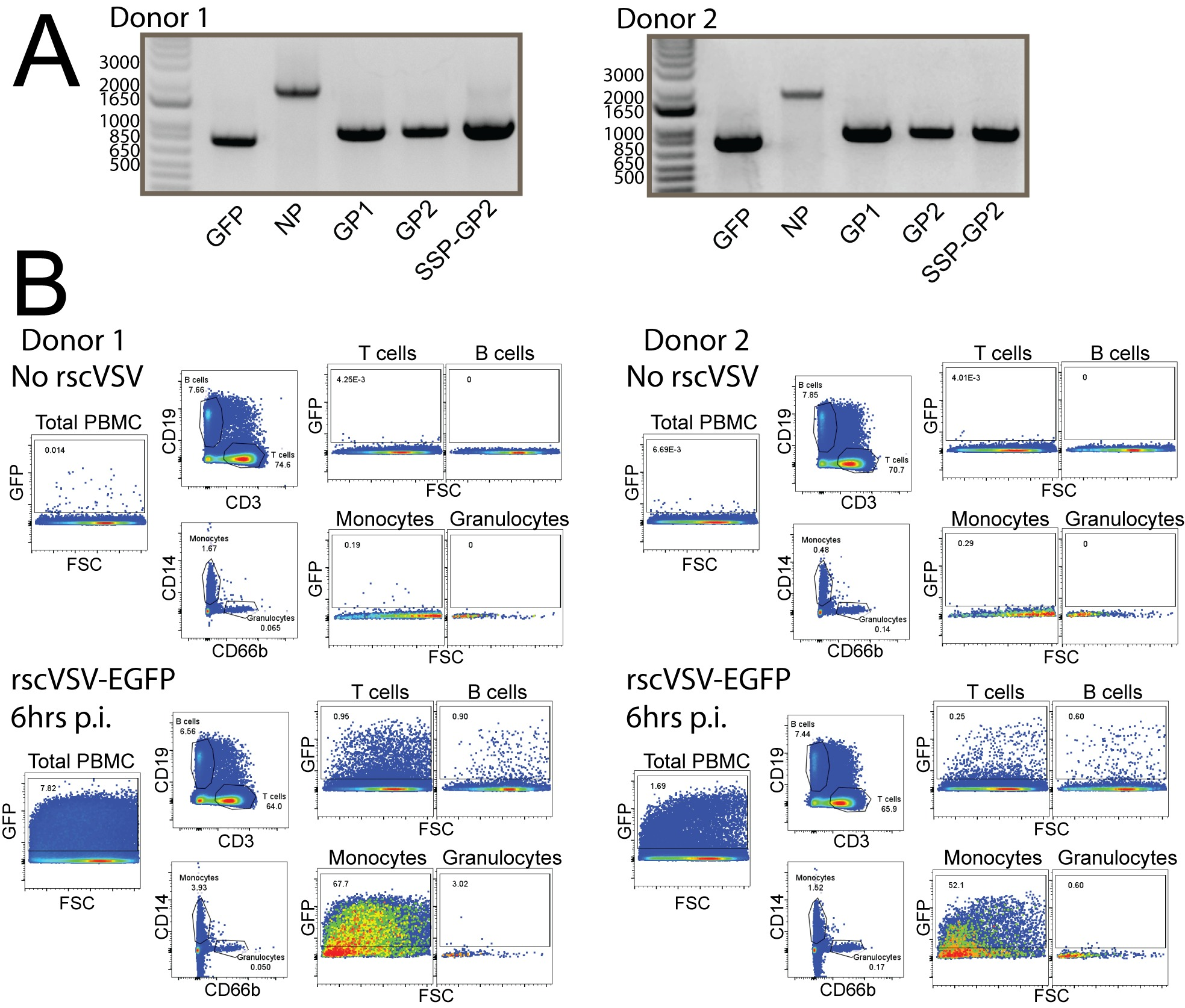

Supplement: S1 Fig — A) PBMCs from two control donors were infected with rscVSVs encoding for the indicated genes. At 6h post-infection, total RNA was isolated and cDNAs made from mRNA using oligo dT primers. Gene specific primers were used to amplify and assess expression of each gene. B) PBMCs from two control donors were infected with rscVSV encoding for EGPF or mock infected. After 6h post-infection, EGPF expression was assessed by flow cytometry in total PBMCs, T and B cells, granulocytes, and monocytes and compared to mock infected PBMCs. (TIF) [file ppat.1008352.s001.tif]

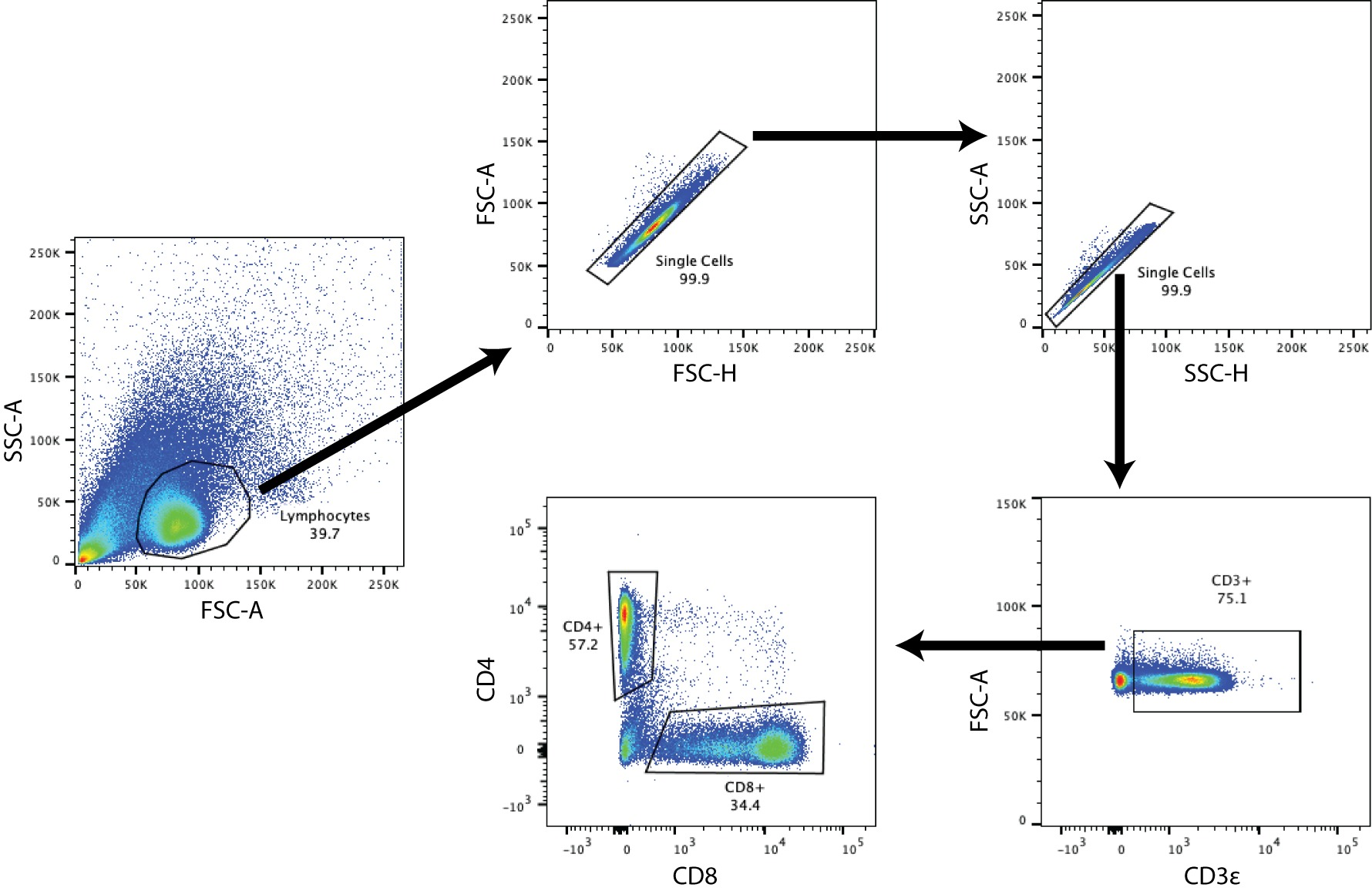

Supplement: S2 Fig — (TIF) [file ppat.1008352.s002.tif]

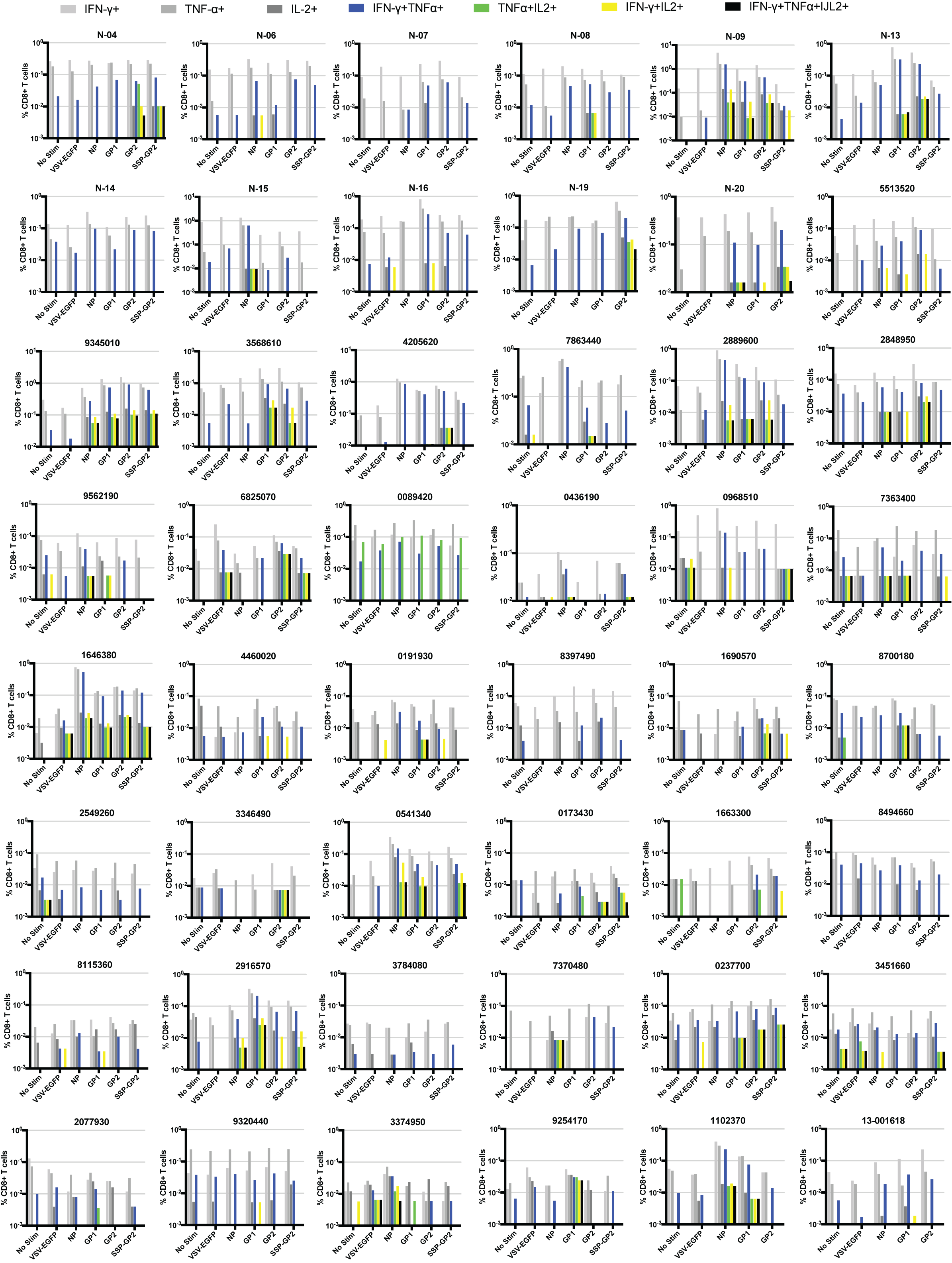

Supplement: S3 Fig — Positive gates were defined as 1.2 log10 fluorescence over the median negative control fluorescence as depicted in Fig 4A and 4B. (TIF) [file ppat.1008352.s003.tif]

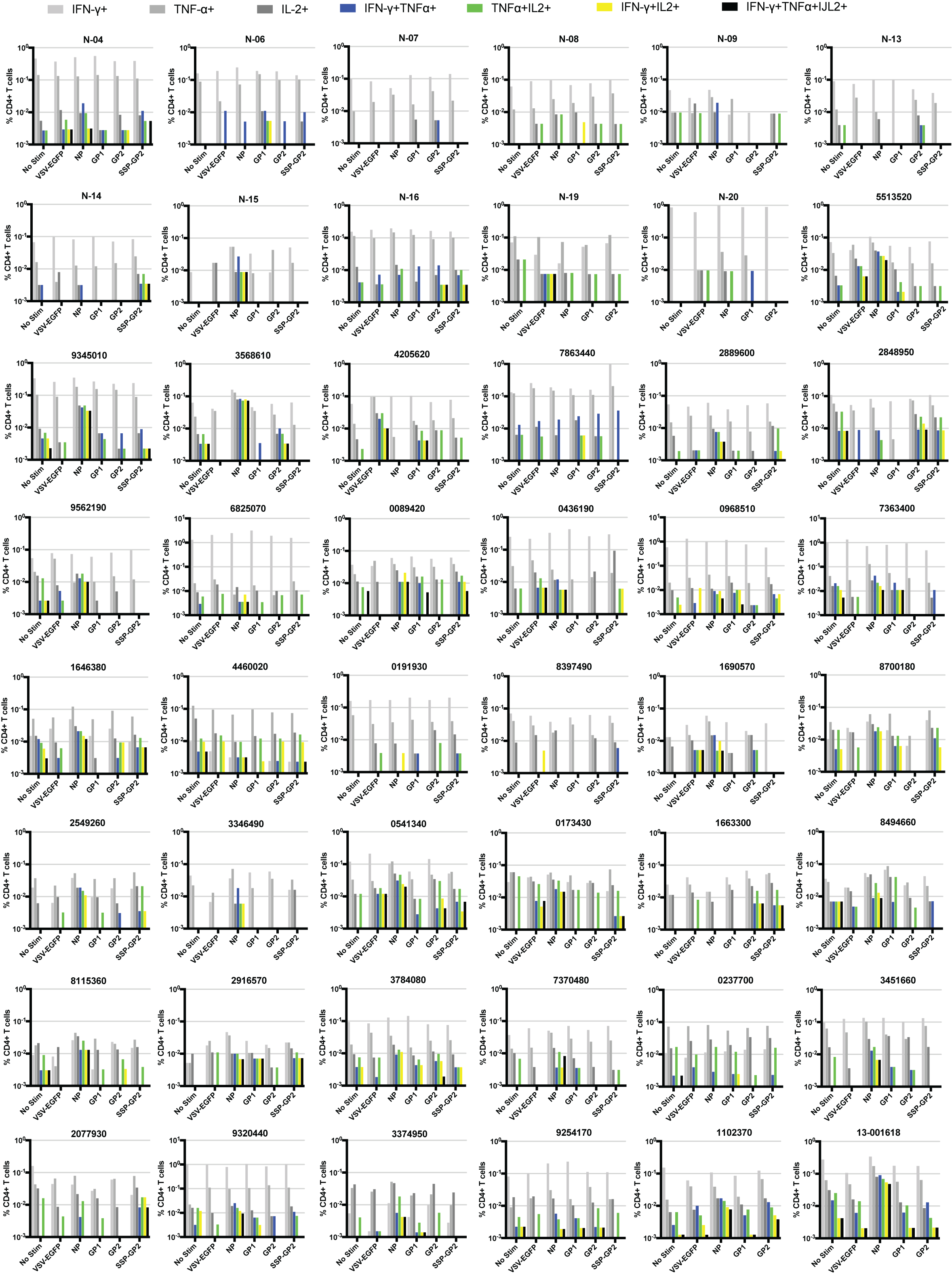

Supplement: S4 Fig — Positive gates were identical to those used for CD8+ T cells. (TIF) [file ppat.1008352.s004.tif]

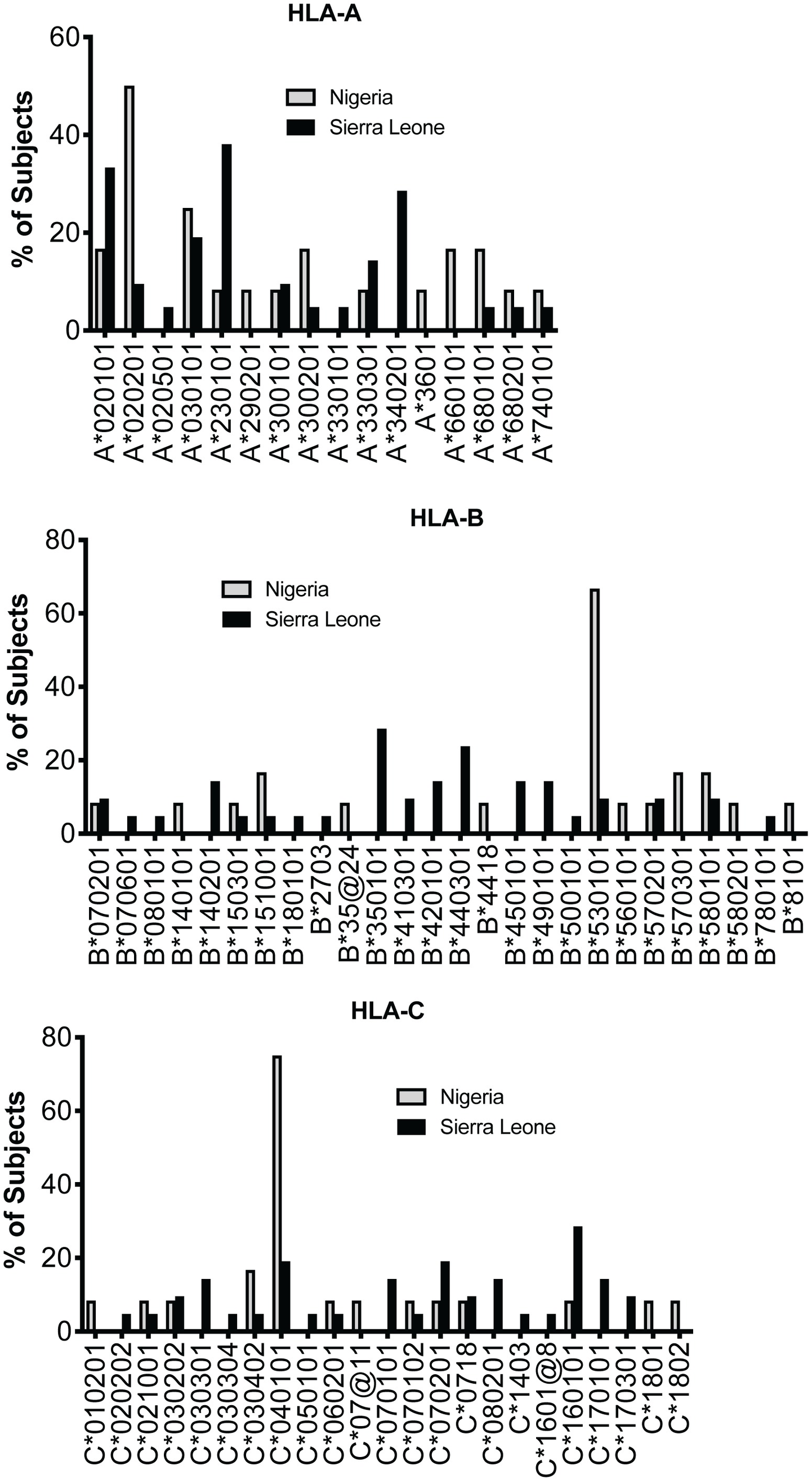

Supplement: S5 Fig — (TIF) [file ppat.1008352.s005.tif]

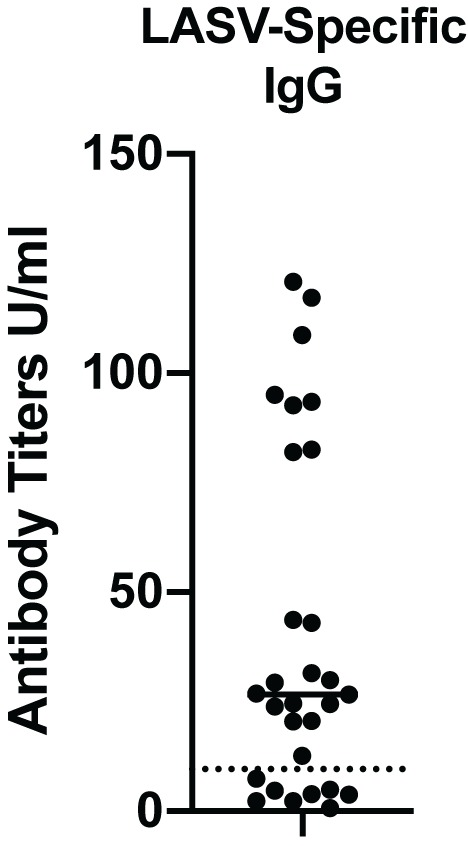

Supplement: S6 Fig — Dotted line represents negative control value. Optical density values for an additional seven patients were obtained but could not be converted into U/ml. However, six of seven were considered positive based on negative control values. (TIF) [file ppat.1008352.s006.tif]
