# Supplementary material for: High crossreactivity of human T cell responses between Lassa virus lineages
Source: PLoS Pathog. 2020 Mar 6;16(3):e1008352. doi: 10.1371/journal.ppat.1008352 (PMC7080273; doi:10.1371/journal.ppat.1008352)
Supplement: S1 Table — Lines in blue indicate a start codon (ATG) was added to the sequence. (PDF) [file ppat.1008352.s007.pdf]

Antigen		Position	# of aa
Full Length	EGFP	1-238	238
	NP	1-569	569
	GP1	1-279	279
	GP2	214-491	278
	ssp-GP2	1-59; 260-491	291
Truncated NP	NP-f1	1-59	59
	NP-f2	40-98	59
	NP-f3	79-145	67
	NP-f4	126-185	60
	NP-f5	166-225	60
	NP-f6	206-265	60
	NP-f7	246-310	65
	NP-f8	291-350	60
	NP-f9	331-390	60
	NP-f10	371-430	60
	NP-f11	411-476	66
	NP-f12	457-527	71
	NP-f13	508-569	62
Truncated GPC	GPC-f1	1-58	58
	GPC-f2	34-93	60
	GPC-f3	75-134	60
	GPC-f4	115-174	60
	GPC-f5	153-212	60
	GPC-f6	194-259	66
	GPC-f7	240-299	60
	GPC-f8	284-343	60
	GPC-f9	322-381	60
	GPC-f10	359-418	60
	GPC-f11	404-463	60
	GPC-f12	445-491	47
