# Supplementary material for: High crossreactivity of human T cell responses between Lassa virus lineages
Source: PLoS Pathog. 2020 Mar 6;16(3):e1008352. doi: 10.1371/journal.ppat.1008352 (PMC7080273; doi:10.1371/journal.ppat.1008352)
Supplement: S2 Table — (PDF) [file ppat.1008352.s008.pdf]

Subject & HLAs	Deduced Epitope	Peptide Sequence	Peptide Position
**N-14**<br><br>A*020101<br>A*030101<br>B*151001<br>B*570201<br>C*030402<br>C*1802	NP 139-172	ALLNMIGMSG<br>GMSGGNQGAR<br>AGRDGVVRVW<br>VWDVKNAELL	NP 139-148<br>NP 145-154<br>NP 155-164<br>NP 163-172
**5513520**<br><br>A*230101<br>A*330301<br>B*450101<br>B*580101<br>C*030202<br>C*040101	GPC 412-451	EMLQKEYMER<br>YMERQGKTPL<br>PLGLVDLFVF<br>VDLFVFSTSF<br>FVFSTSFYLI<br>FSTSFYLISI<br>TSFYLISIFL<br>FYLISIFLHL	GPC 413-422<br>GPC 419-428<br>GPC 427-436<br>GPC 431-440<br>GPC 434-443<br>GPC 436-445<br>GPC 438-447<br>GPC 440-449
