# Supplementary material for: High crossreactivity of human T cell responses between Lassa virus lineages
Source: PLoS Pathog. 2020 Mar 6;16(3):e1008352. doi: 10.1371/journal.ppat.1008352 (PMC7080273; doi:10.1371/journal.ppat.1008352)
Supplement: S3 Table — (PDF) [file ppat.1008352.s009.pdf]

Construct	Expected length	Forward Primer
EGFP	757	TATCACGCGTGGTATGGTGAGC
NP	1747	TATCACGCGTGGTATGAGTGCCT
GP1	877	TATCACGCGTGGTATGGGACAAA
GP2	874	TATCACGCGTGGTATGACTAGTT
SSP-GP2	916	CGATCTGTTTACGCGTATGGGACAAATAGTGACATTCTTCCAGGAA
NP f1	220	CGATCTGTTTACGCGTATGAGTGCCTCAAAGGAAATAAAATCCTTT
NP f2	223	CGATCTGTTTACGCGTATGCATGGACTTGACTTCTCCGAAGTCAGTAAT
NP f3	247	CGATCTGTTTACGCGTATGGAATTAAAATCAACTCAACAAAAGAGTATACTGAGAGTT
NP f4	223	CGATCTGTTTACGCGTATGGGCAACCTAAGCTCACAGCAA
NP f5	226	CGATCTGTTTACGCGTATGGTGAAAAATGCAGAGTTGCTCAATAATCAG
NP f6	226	CGATCTGTTTACGCGTATGGGTTTGATCTACACAGCAAAGTATCCCAA
NP f7	241	CGATCTGTTTACGCGTATGTTTAGCTTGGGTGCAGCTGTGAAGG
NP f8	223	CGATCTGTTTACGCGTATGTTCATTTCAGACACCCCTGGTGA
NP f9	226	CGATCTGTTTACGCGTATGTGGGAAAACACTGTCGTTGATCTGG
NP f10	223	CGATCTGTTTACGCGTATGACCCTCAAGGATGCAATGCTGCAACTT
NP f11	244	CGATCTGTTTACGCGTATGATACACTTCTTCCGTGAACCTACTGATTTAAAG
NP f12	256	CGATCTGTTTACGCGTATGGTCATTACCTGTCAGGGGTCCGATGAC
NP f13	229	CGATCTGTTTACGCGTATGCACACAGGTGTCGTTGTTGAAAAGAAG
GPC f1	217	CGATCTGTTTACGCGTATGGGACAAATAGTGACATTCTTCCAGGAA
GPC f2	226	CGATCTGTTTACGCGTATGGGTCTGTACAATTTTGCAACGTGTGGCCTT
GPC f3	223	CGATCTGTTTACGCGTATGGAGACACTCAATATGACCATGCCTCT
GPC f4	226	CGATCTGTTTACGCGTATGCACAAATTTTGCAATCTGTCTGATGCCCA
GPC f5	223	CGATCTGTTTACGCGTATGAGCTGCGATTTTAATGGGGGAAAGAT
GPC f6	241	CGATCTGTTTACGCGTATGGCTTGGGGTGGGAGCTACATTGCTC
GPC f7	226	CGATCTGTTTACGCGTATGGGTTATCTCGGGCTCCTCTCACAAAGGAC
GPC f8	223	CGATCTGTTTACGCGTATGCTAATTGAGGCTGAACTAAAATGCTTC
GPC f9	226	CGATCTGTTTACGCGTATGGCCATTCAAAGGTTGAAAGCTGAAGCACA
GPC f10	223	CGATCTGTTTACGCGTATGGGAATTCCATACTGTAATTACAGCAAGTATT
GPC f11	226	CGATCTGTTTACGCGTATGGAACAACAAGCTGACAATATGATCACTGAGA
GPC f12	187	CGATCTGTTTACGCGTATGATCTTCCTTCACCTAGTCAAAATACCAACTCA
		**Reverse Primer for all constructs**
		TCATTTGTCGTCGTCGTCTTTGTAGTC
