# Supplementary material for: High crossreactivity of human T cell responses between Lassa virus lineages
Source: PLoS Pathog. 2020 Mar 6;16(3):e1008352. doi: 10.1371/journal.ppat.1008352 (PMC7080273; doi:10.1371/journal.ppat.1008352)
Supplement: Attachment — Submitted filename: PLOSP Response.docx [file ppat.1008352.s010.docx]

Reviewer #1: Sullivan et. al., are seeking to determine the nature of T-cell response in survivors of Lassa fever. To do this they used recombinant single cycle Vesicular Stomatitis Virus (rscVSV)-G pseudotyped viruses, encoding complete or partial fragments of the Lassa virus (LASV) (lineage IV) nucleoprotein (NP) and glycoprotein precursor protein (GPC), to infect PMBCs from Lassa fever survivors from both Nigeria and Sierra Leone (LASV, presumably lineages II and IV). They then measured CD4+ and CD8+ T cell responses in the VSV-infected PBMCs of the survivors and found that all survivors were able to mount T-cell responses towards lineage IV proteins, regardless of their country of origin. This suggests that lineages may share T-cell epitopes that might be exploited for vaccine targeting. However, there are significant reservations relating to the underlying method being applied to measure T cell responses, the absences of key pieces of data from figures, and the lack of confirmatory analysis using peptide-based assays. The net result is that it is difficult to determine the validity and impact of the results without more experimental data being provided.

We thank the reviewer for the thorough review. We have provided the reviewer with additional data and hope that this is sufficient to allay concerns about the validity of our methods. Importantly, while perhaps less sensitive than other methods (T cell expansion, T cell clones, peptide stimulation over longer periods), our short rscVSV-mediated stimulations retain the processes of antigen processing and presentation that is critical for the selection or relevant peptides. This process is lost during direct peptide stimulation and could lead to false positive results as TCRs have been shown to respond to peptides similar to its cognate peptide. We would like to bring to the reviewer’s attention several studies showing that TCRs can respond to significant number of different peptides (Birnbaum et al, Cell, 2014; Sharma et al, Cancer Immunol Res., 2019; Wooldridge et al, JBC, 2012; Harndahl et al, EJ Imm, 2012). While there is certainly an important role for peptide stimulation assays, and many epitopes have been discovered in this manner, the method is not without its own issues, as illustrated by Assarsson et al (J Immunology, 2007) showing that “*of all peptides that bind MHC, are immunogenic, and are generated by Ag processing, only 1 of 10 is actually recognized after VACV infection*”.

In our studies, we have considered that using a more conservative approach of presenting antigens through viral infection would be preferable for several reasons. 1) Developing countries are antigen rich environments that shape the immune system very early in life (Wagar et al, Front Imm., 2019), and limiting the peptide pool to those that are processed and presented naturally could limit potential false positive hits. 2) HLAs have been poorly defined in our patient population. Our unpublished studies of nearly 400 individuals, revealed that 16% of alleles we have sequenced have not been described or entered into the IMGT database. Therefore, peptide pools based on HLA binding to “common” HLAs could have missed T cell responses to important epitopes. 3) We were limited by the number of cells we could obtain from each survivor. With only 3 x 10^7^ cells (average) per individual, we could not perform the deconvolution of peptide matrices that is necessary to identify epitopes and further validated them through other methods. We found that by comparing T cell responses to whole antigens, small ~60aa portions of those antigens, and finally using peptides derived from these smaller sequences, we were able to compare peptide to whole antigen and 60aa polypeptide responses while minimizing false positive responses in areas that did not show reactivity with 60aa polypeptides. This could all be accomplished with the small amount of sample we were able to work with.

To address the concerns of the reviewer we have revised our manuscript with the following major changes: 1) Added figure 4 containing new data on two 10aa peptide epitopes derived from deduced epitope sequences; 2) Added figures S3 and S4 showing single, double, and triple cytokine expression levels for CD4+ and CD8+ T cells stimulated with rscVSVs encoding whole LASV antigens; 3) Added a new analysis of amino acid conservation; 4) Added language addressing methodology concerns in the discussion and 5) deposited all flow cytometry data in a publicly-accessible online flow cytometry database.

Reviewer #2: Sullivan et al provide a collection of data supporting the cross clade responses of T-cells as observed from LASV survivors from both Nigeria and Sierra Leone. The study has immediate importance as the sample populations are from humans and thus has immediate relevance with respect to interpretation; however, is not without caveats associated with this kind of study (ie unknown time since or severity of infection, length of convolescence, ect...) of which the authors adequately recognize. The major findings suggest conserved T-cell epitopes common across phylogeographic regions and isolates, a finding rightly recognized as novel. While there are also caveats in the methodology utilized, I believe the authors adequately addressed the shortcomings (i.e. use of recombinant VSV versus other methods). This work will be of great interest to those studying arenavirus immunology, specifically those interested in old world arenavirus infections as well as those interested in developing vaccines against these high priority agents.

We concur with the reviewer that our methodology does have drawbacks and lacks sensitivity. However, we think that our methods have a distinct advantage (explained above) especially given our focus in this manuscript on identifying dominant CD8+ T cell responses in survivors in a large cohort of individuals.

Reviewer #3: Lassa virus is the causative agent of Lassa Hemorrhagic Fever (LF), with high morbidity and mortality in West Africa.  Currently, four lineages of the Lassa virus circulate in West Africa, although only lineage IV is the main target in the most current vaccine candidates. Therefore, It is important to assess if the Lassa-specific immune response is able to cross-recognize antigens deriving from the four different lineages. Following this concept, in this manuscript, Sullivan et al. sought to determine the memory T cell reactivity against Lassa lineage IV antigens in Lassa infected survivor from Nigeria (where only lineages I-III circulate) and Sierra Leone (where only lineage IV circulate).  The authors found no difference in terms of T cell reactivity between Nigerian and Sierra Leone infected survivors, suggesting that Nigerian memory T cells are able to cross-recognize lineage IV with a magnitude of response comparable to the ones observed in Sierra Leone survivors where only lineage IV circulation is observed. These results have important implications in Lassa vaccination design however, a more in-depth characterization of T cell reactivity, as well as additional information on the specific epitopes, is required.

We thank the reviewer for her/his positive comments. We have added additional data into the revised paper to address the reviewer’s concerns. See comments to Major Issues.

**Part II – Major Issues: Key Experiments Required for Acceptance**

Reviewer #1: Specific concerns:

1. To determine T cell responses to Lassa antigens, the experiments presented utilize recombinant single cycle Vesicular Stomatitis Virus (VSV) pseudotyped with its native glycoprotein, G, added in trans. The genomes of these viruses have had the gene for G replaced with the Lassa NP, NP fragments, GPC, GPC fragments or GFP. In the first experiments, viruses encoding larger gene fragments were individually incubated with PBMCs from survivors for four hours, followed by the addition of brefeldin A (~16 hours) and then the cells were fixed, permeabilized and CD4+ or CD8+ T cell intracellular cytokine staining for TNF-a and IFN-g was measured using flow cytometry. Subsequent experiments used smaller overlapping gene fragments to “map” the T cell epitopes. There are several concerns about the use of this method and the control data that are presented:

a. The assay system uses bulk PBMCs infected by the recombinant single cycle VSV (rscVSV) viruses to present Lassa antigens via MHC. However, there is no evaluation of the ability of rscVSV to transduce PBMCs or the types of cells that are being transduced. Instead transduction, transcription and translation of Lassa proteins is shown in the BHK-21 kidney cell line (see Figure 1B). This cell line is highly permissive to VSV infection and are often used for the generation of pseudotyped VSV virus. Since efficient transduction of antigen presenting cells is a key component of the assay, they should show transduction, transcription and protein expression in human PBMCs samples. Ideally this would be done for each survivor, but given the likely limited amount of PBMCs available, it is reasonable to perform this analysis on several naïve human samples to show consistency of transduction, transcription and expression levels between subjects.

Our experimental approach was based on one that has been well characterized (Moseley, Immunol Methods, 2012). VSV is monocyte and dendritic cell tropic with little to no infection of T, B or NK cells. As monocytes are a small percentage of PBMCs, we chose to show mRNA and protein expression in a more permissive culture (Figure S1A). Even in a more permissive culture, we were unable to identify protein expression in a majority of our constructs (GP2 and all overlapping ~60aa polypeptides). While stable protein expression is likely important for MHC class II presentation, it is absolutely not required for MHC Class I presentation and, in fact, rapid degradation may facilitate presentation of LASV peptides encoded by our rsvVSVs.

Similar to the paper by Moseley et al, we found that the majority of CD14+ monocytes were infected with a rscVSV encoding GFP, whereas T cells were not infected to an appreciable amount (<1% after 6 hours) (Figure S1B).

b. In each of the figures with T-cell responses, there is no representation of the “no stimulation” and the negative control (GFP virus) on the charts. Similarly, there are no representations of the flow cytometry images for the figures. These data are critical to the interpretation of the data and to determine the validity of the analysis. These controls and panels were provided in the authors previous PNAS manuscript on Ebola (PNAS. 2018. 115(32:e7578-86) and should be provided in this manuscript as well.

We observed that some individuals had some background activation which would have made comparisons between individuals inaccurate if only the raw, non-normalized values were used. Accordingly, we presented our data subtracting background of negative controls (unstimulated or rscVSV-GFP stimulated). Although this is mentioned briefly in the figure legend to figure 3 (lines 725-727) we have added similar language to the results section (Lines 204-207) to make this clearer to the reader.

In addition, we have added Figures S3 & S4 showing non-normalized values for CD8+ and CD4+ T cell responses to rscVSVs encoding LASV antigens in all 48 individuals in this study. These figures include percentages of single, double, and triple cytokine expressing cells. We have also added two examples in a new Figure 4, showing responses to rscVSVs encoding whole antigens, ~60aa fragments, and 10aa peptides. Lastly, we are submitting all our flow data to the Flow Repository Database so that our raw data can be evaluated if needed. We have added language to the Methods section to indicate where our data has been deposited under the subheading “Data Sharing” (lines 654-659)

c. Similarly, since the results and the analysis are looking at very low frequency events, gating strategies can have a significant impact on the results. In addition to the information requested in 1b (above), please provide a supplemental figure showing representative gating trees for each of the unique strategies used.

Gating is shown in Figure S2. Gating of cytokine expressing cells are shown in Figure 4.

d. The multiplicity of infection (moi) of 15 is very large and means that >99.5% of the PBMCs would be infected more than one time, including T cells (J Exp Med. 1978. 148(4):837-49). Given the critical dependence of the T-cell assays on both T-cells and APCs, it is important to demonstrate which cell types are being infected and to what extent. This analysis could be performed on several naïve human samples using rscVSV-GFP pseudotype viruses.

The rscVSVs are tittered on BHK-21 cells, and the MOI used to infect PBMCs was chosen to generate optimal T cell responses. We and others have shown that most cells in the PBMC culture are not permissive to rscVSV infection. As explained in our response to Major Concern 1b, we have added Figure S1B with data consistent with that in Moseley et al.

e. Despite there being staining for TNF-a, IFN-g and IL-2 by intracellular staining, data is only shown for magnitude of TNF-a/IFN-g double positive cells. Data on the magnitude and proportion of single, dual and triple positive intracellular cytokine responses should be discussed and presented, even if they were not significantly different than the GFP control virus.

As discussed in our response to Major Concern 1b, we have added Figures S3 & S4 showing percentages of single, double, and triple cytokine expressing cells. We have briefly discussed these data in the manuscript in lines 355-362

2. Putative T-cell epitopes are identified as candidates and there is no confirmation using peptide-based assays. This is an important confirmation that should be performed, and the data provided in a main figure. Also, there is a lack of a discussion/figures showing the locations of the identified putative epitopes, their conservation between lineages and what the overlap or lack of overlap means in the context of next steps for a vaccine. It would helpful to provide this information in the text and a figure to accompany the analysis that shows the sequences from the lineages I-III and their alignment with the lineage IV sequence identified in this study.

While we believe that identifying minimal peptide epitopes is outside the scope of this study, we acknowledge that “deduced epitopes” are not strictly experimentally defined and that the deduction may fall short in some cases. Specifically, it is possible that similar responses to adjacent 60aa polypeptides could mean that there are two epitopes in non-overlapping regions that produce similar T cell responses. We have added this caveat to the discussion in lines 373-377. To allay some of the concerns of the reviewer, we have included two peptide epitopes found using sequences from deduced epitopes and HLAs from the same individuals harboring those epitopes to identify candidate 10aa peptide epitopes for testing. These peptides were experimentally tested and found to be present to comparable levels as rscVSV responses to these regions (Figure 4). Methods, Results, and Discussion section have been altered to include these new data.

We have also performed a genetic analysis demonstrating the conservation of amino acid sequences in regions that harbor the most CD8+ T cell epitopes and discussed in the Results and Discussion sections.

Reviewer #2: None, the study was well done.

Reviewer #3: T cell reactivity is determined analyzing only polyfunctional T cells IFNγ+TNFα+ double producers, however, it is not clear if these polyfunctional T cells represent the majority of T cell responses, or if the instead stronger magnitude of responses is observed when looking at single IFNγ or TNFα producer.

Does the frequency of responders increase if the single producers are considered?

Additionally, in the methods, authors also stain for IL-2 production but do not show any data relating to this issue and do not report whether they the observe triple producer T cells.

In mouse experiments where naïve animals have minimal background expression of these cytokines, it is important to take single producers into consideration. However, in human PBMCs due to the variable and high background signal of “single producers” we only consider double producers and these were unique to LASV antigen stimulated PBMCs. Because IL-2 expression was consistently lower than IFN gamma and TNF alpha expression and therefore, we used double IFN gamma and TNF alpha expression as an indication of T cell activation. However, in order to show a more complete dataset, we added data for single, double, and triple cytokine expressing cells in Figures S3 & S4.

Authors dissect T cell reactivity by using fragments spanning 60aa length, and they deduce CD8 epitopes by identifying as epitopes the overlapping region. This approach is not suitable to define the CD8-T cell epitopes with sufficient accuracy. I would suggest either removing this analysis or, based on mapping the potential epitopes at least in silico by the combined use of HLA typing information and bioinformatic predictions.

We had previously found that smaller 10aa epitopes are consistently found in our deduced epitopes. However, we acknowledge, and as discussed in responses to Issue #2 from Reviewer 1, deduced epitope analysis may not accurately capture epitope containing regions in all instances. We add moderating language (lines 373-377) to address these concerns. We also added data where were identified two peptide epitopes within deduced regions in a new Figure 4.

**Part III – Minor Issues: Editorial and Data Presentation Modifications**

Please use this section for editorial suggestions as well as relatively minor modifications of existing data that would enhance clarity.

Reviewer #1: Other concerns to address:

3. Many in the field are using overlapping bulk peptides and peptide pools to map CD4+ and CD8+ T-cell epitopes. Despite being more costly, use of peptides avoids the confounding effects of using viruses that are capable of inducing cytokine responses and infecting all the cells being used in the assay, including T cells. Please provide a few sentences on why the rscVSV approach was chosen, it’s limitations and benefits compared to the peptide method.

4. Abstract: Line 45-46 mentions four viral lineages with substantial inter- and intra-lineage diversity. However, there is substantial inter-lineage diversity, but the intra-lineage diversity isn’t considered to be substantial (Ehichioya DU et al. Phyelography of Lassa virus in Nigeria. J Virol. 2019.93(21)). In fact, the idea that underlies the notion of having a single vaccine that is protective against all the lineages rests on the inter-lineage diversity.

We agree with the reviewer that a major concern with a LASV vaccine is protection across lineages and not within lineages. Accordingly, we have modified the abstract and removed intra-lineage diversity. However, several papers we cited in the discussion (Leski et al (33) and Oloniniyi et al (34)) specifically identify enough variation within lineages to suggest classification into sub-lineages or clades. Leski et al, suggest subclassification of lineage IV into 5 separate clades, with a mean amino acid diversity of 7% for the nucleoprotein. There is also a more accepted classification of lineage two into two subclades, A and B based on sequence diversity. Lastly, a study of 77 sequenced strains (Ehichioya et al, Journal of Virology, 2019), identified seven sub-lineages in lineage II and five in lineage III. Because of these findings, we kept a sentence in the discussion related to intra-lineage diversity (line 308).

5. On page 6 (lines 109 and 100) the number of subjects tested are 11 Nigerians and 37 Sierra Leoneans (total of 48). On lines 160 through 162, the authors describe the responses in these 48 individuals as being made up of 16 with both CD4 and CD8 responses, 13 with just CD4 responses and 20 with just CD8 responses (total = 49). Please correct the numbers.

We apologize for the inconsistency. There were 19 survivors with measurable CD8 responses. This has been corrected in both the manuscript and in figure 2A.

6. Introduction: Line 86 – 87. Recommend changing “…the western edge of continental Africa to Nigeria” to “West Africa” as what is being described is more properly referred to as West Africa.

We have made this change.

7. Results: Line 126 LCMV should be changed to LASV.

We have made this change.

8. Line 137 Fig 1A & 1C. There was no label for lane 4.

We have replaced “lane 4” with “SSP-GP2” so it is more readily identifiable.

9. Line 171. “…LASV strains from lineages II and III.” The manuscript assumes the strains of the survivors were likely lineages II and III. However, current literature on the molecular epidemiology of LASV shows that LASV strains in Irrua where the Nigerian survivors were from, are lineage II strains [Ehichioya DU et al. Phyelography of Lassa virus in Nigeria. J Virol. 2019 Oct 15;93(21)]. Thus, in the absence of confirmed LASV sequences matched to each survivor, it is best to assume the survivors were infected by lineage II viruses.

The paper the reviewer referenced was a study performed on samples from patients seen at the Irrua Specialist Teaching Hospital (where our samples also originate). In this study, they identify samples from both lineage II and III viruses. LASV lineage III is present north of Irrua. However, the ISTH is a well-established reference hospital for LF, receiving patients from a large geographical swath of Nigeria, especially during the time these survivor samples were collected (2014). While most of the viruses sequenced in Ehichioya et al (Phylogeography of Lassa Virus in Nigeria. Journal of virology. 2019) were of lineage II origin, a substantial number of Lineage III sequences were recovered (see Figure S3 and S4 in Ehichioya et al). We have added a clarifying sentence (lines 152-155) describing these published results.

10. Line 163, 165, 168 and Figure 2B. In these lines and figure panel, the manuscript shows a combined NP & GPC. It is not clear whether this is (1) the result of two separate PBMC infections added together OR (2) if PBMCs were infected simultaneously with rscVSV NP and rscVSV GPC

We added two sentences in the figure legend for Figure 2 that helps clarify how we classified samples that responded to GPC and NP.

11. Materials & methods:

a. Residue numbers, cloning or synthesis method and other details specific to the vector production should be provided in the section on the rscVSV preparation (line 330)

Additional information relating to construction of the rscVSV rescue constructs was added to the Methods section.

b. Line 337. Ebola should be changed to LASV.

We have made this change.

c. Line 344. The forward primer sequences used for the PCR should be provided and the expected band size of the amplicon stated.

We have added Table S3 with this information.

12. Figure 1A. It is unclear why there is a schematic for the entire L and S segments, would consider removing.

We added this figure to give the reader an idea of the relative sizes of the LASV genes and the overall genomic organization to put the gene product schematics presented below into context.

13. S.P should be S.S.P (stable signal peptide).

We have made this change.

14. Figure 1B & C. While size markers are shown, there are no values given for the markers. Please label 3-4 of the major bands of the DNA and protein ladders, particularly those near bands of interest.

We have made this change.

Reviewer #2: Lines 100-102: Consider revising or removing this sentence as it is not in the same context as natural infection. Vaccinia delivery of antigen is an artificial system that is not the same context as natural arenavirus infection. The importance of cell mediated immunity can be better argued another way, even using LCMV as a surrogate would be more appropriate. VSV delivery of LASV antigens induces a neutralizing antibody response (<https://journals.plos.org/plosmedicine/article?id=10.1371/journal.pmed.0020183#s3>), but at the same time does not imply the importance of humoral immunity in clearance of natural LASV infection, only that protection is possible via the context of VSV infection where LASV GPC is the surrogate glycoprotein. Please correct this.

We concur with the reviewer and apologize for conflating acute and protective responses. We have separated protective responses in a separate paragraph, adding both the important study cited by the reviewer and another study showing that protection can be delivered through monoclonal antibody therapy.

The authors suggest a conserved region(s) of LASV antigens that both survivors from Sierra Leone and Nigeria recognize. Readers might benefit from knowing what level of phylogenetic conservation at nucleotide or amino acid level exist across clades in these "hot spot regions". Please consider including this to support your conclusions.

We thank the reviewer for this suggestion and have added language in the results (lines 285-301) and discussion (Lines 379-387)

Reviewer #3: Figure 3C is not discussed in the results section.

We have made this change.
